# A295 A CHALLENGING DIAGNOSTIC CASE - SEVERE ACUTE HEPATITIS POTENTIALLY ASSOCIATED WITH COVID-19 IN AN IMMUNOCOMPETENT YOUNG ADULT

**DOI:** 10.1093/jcag/gwad061.295

**Published:** 2024-02-14

**Authors:** B Lawendy, D Hudson, R Abdul-Karim

**Affiliations:** Western University, London, ON, Canada; Western University, London, ON, Canada; Western University, London, ON, Canada

## Abstract

**Background:**

Severe hepatitis with liver enzymes in the thousands is usually associated with viral etiologies, toxins, vascular aetiologies, acute gall stones, autoimmune hepatitis, and Wilson disease. Acute hepatitis in the moderate range has been associated with COVID19 infection.

**Aims:**

A potential case of severe hepatitis with initial ALT of 6863, who tested positive for COVID19. Acetaminophen ingestion confounded the presentation.

**Methods:**

Case Report

**Results:**

A 48-year-old woman, with history of Insomnia on Zopiclone, presented to Hospital with a 6-day history of fatigue, malaise, muscle aches and subjective fevers. She had a ten pack-years smoking history, consumed 8 alcoholic beverages weekly, used marijuana infrequently, and denied any herbal or naturopathic supplement usage. She was double vaccinated against COVID19 over a year ago.

On the 2^nd^ day of symptoms, she ingested 5 grams of Acetaminophen over a 24-hour period. Subsequently, she continued to use Acetaminophen, but did not exceed a daily dosage of 4 grams. On the 5^th^ day, she presented to clinic and was found to have elevated liver enzymes: ALT 6863, AST 5785, ALP 62, GGT 60, and INR 1.1 with Bilirubin 8. She had nausea and emesis, prompting her to present to the hospital on the 6^th^ day. The patient was alert and oriented, with mild tenderness in the right upper quadrant with no jaundice, ascites, peripheral edema, or encephalopathy.

She had reactive lymphocytosis consistent with a potential viral infection. Testing for acute hepatitis A, B, and C, HIV, EBV, CMV, herpes simplex virus, and varicella-zoster was negative. COVID19 was detected by rapid antigen testing and confirmed via PCR. Blood and urine toxicology screens were unremarkable. Acetaminophen was detected at a low level of 67 μg/mL at presentation and given the diagnostic dilemma & concern for concomitant acetaminophen-associated liver injury she was started on N-acetylcysteine (NAC) infusion. Acetaminophen level was trended until undetectable. Ceruloplasmin and alpha-1 antitrypsin were unremarkable. Iron studies showed an elevated Ferritin 1032. Autoimmune workup was negative except for anti-dense fine speckled autoantibodies with 1:80 (low) titre. Abdominal ultrasound with doppler was normal.

Her liver enzymes improved (Figure1) without signs of acute fulminant liver failure. She was discharged after 48 hours (day 7). In follow-up, her labs were: ALT 11, AST 16, INR 1.1, Bilirubin 6, and Ferritin 61.

**Conclusions:**

To our knowledge, this is one of the first case reports of severe hepatitis, possibly due to COVID-19 in an immunocompetent adult, however, concerns for concomitant unintentional acetaminophen toxicity complicate the diagnosis. NAC was given to mitigate diagnostic error given the risk of anchoring bias. If patient condition had deteriorated liver biopsy would have been considered.

**Funding Agencies:**

None

